# A catalog of stability-associated sequence elements in 3' UTRs of yeast mRNAs

**DOI:** 10.1186/gb-2005-6-10-r86

**Published:** 2005-09-30

**Authors:** Reut Shalgi, Michal Lapidot, Ron Shamir, Yitzhak Pilpel

**Affiliations:** 1 Department of Molecular Genetics, Weizmann Institute of Science, Rehovot, 76100, Israel; 2 School of Computer Science, Tel-Aviv University, Tel-Aviv, 69978, Israel

## Abstract

**Background:**

In recent years, intensive computational efforts have been directed towards the discovery of promoter motifs that correlate with mRNA expression profiles. Nevertheless, it is still not always possible to predict steady-state mRNA expression levels based on promoter signals alone, suggesting that other factors may be involved. Other genic regions, in particular 3' UTRs, which are known to exert regulatory effects especially through controlling RNA stability and localization, were less comprehensively investigated, and deciphering regulatory motifs within them is thus crucial.

**Results:**

By analyzing 3' UTR sequences and mRNA decay profiles of *Saccharomyces cerevisiae *genes, we derived a catalog of 53 sequence motifs that may be implicated in stabilization or destabilization of mRNAs. Some of the motifs correspond to known RNA-binding protein sites, and one of them may act in destabilization of ribosome biogenesis genes during stress response. In addition, we present for the first time a catalog of 23 motifs associated with subcellular localization. A significant proportion of the 3' UTR motifs is highly conserved in orthologous yeast genes, and some of the motifs are strikingly similar to recently published mammalian 3' UTR motifs. We classified all genes into those regulated only at transcription initiation level, only at degradation level, and those regulated by a combination of both. Interestingly, different biological functionalities and expression patterns correspond to such classification.

**Conclusion:**

The present motif catalogs are a first step towards the understanding of the regulation of mRNA degradation and subcellular localization, two important processes which - together with transcription regulation - determine the cell transcriptome.

## Abstract 2

By analyzing 3' UTR sequences and mRNA decay profiles in yeast, 53 sequence motifs have been identified that may be implicated in stabilization or destabilization of mRNA.

## Background

In recent years, the *de novo *computational discovery of regulatory sequence motifs has advanced tremendously due to the integration of large-scale data, predominantly on genomewide gene expression. Correlations between presence of sequence motifs in promoters and particular gene expression profiles are hypothesized [1-5] and occasionally verified [6,7] to be causative of such expression patterns. In contrast, RNA motifs, particularly those residing in 3' untranslated regions (UTRs) of genes, have received less attention so far, and most information comes from individual gene cases. In humans, a regulatory element called ARE (A/U Rich Element), which usually resides in the 3' UTRs of mRNAs, has been identified, and was found to enhance destabilization of the mRNA by directing rapid deadenylation [8,9]. Based on human mRNA decay profile kinetics, Yang *et al*. identified sequence motifs that are enriched in either fast or slow-decaying transcripts [10]. A recent study in humans published a set of 72 highly conserved 3' UTR motifs, half of which are associated with microRNAs [11]. Binding by microRNA, in turn, was shown in some cases to be predictive, and most probably causative, of transcript degradation [12]. On the other hand, the mechanisms mediated by non-microRNA-related motifs are not yet understood.

Despite impressive progress in the ability to model steady-state transcript levels in yeast based on transcription initiation motifs [13], it is clear that complementary understanding of transcript degradation regulation is needed for a complete picture. Yet in contrast to the advances made in mammalian genomes, very little is known about the control of transcript degradation in other species. In the present study we reasoned that computational means that have so far been mainly applied in the analyses of promoter-acting regulatory motifs may be adapted for the discovery of functional motifs in 3' UTRs on a genomewide level. Yet, since the biological effects of such motifs are likely to be inherently different from those related to transcription initiation, the success of such an endeavor critically depends on the existence of high-quality raw data relevant for the role of 3' UTR motifs. Here we present a two-stage process that aims at deriving a catalog of sequence motifs that may affect yeast mRNA stability; the first stage is based on genomewide data on mRNA half-life [14], and the second stage on evolutionary conservation. The analysis resulted in a novel catalog of 53 motifs that are associated with either increased or decreased transcript stability. We estimate that the transcript stability of 35% of all yeast genes is subject to regulation by these motifs.

## Results

### Deriving a stability-associated sequence motif catalog: the first stage

First, we used genome-wide expression data to derive an initial catalog of 3' UTR sequence motifs, which are associated with either significantly increased or decreased mRNA half-lives. We based this stage on data of mRNA half-lives by Wang *et al*. [14], which were derived from mRNA decay profiles measured by microarrays following transcription initiation shut-down. We searched for 3' UTR sequence motifs correlative with extreme half-life values in two ways. In the first method we exhaustively enumerated all possible k-mers and sought significant association between occurrences of a k-mer in the 3' UTR of genes and increased or decreased mRNA half life. In the second method we looked for over-represented motifs within gene sets with particularly low or high half-life values.

### Indexing 3' UTRs of all yeast genes

Using the 'Virtual Northern' data [15], we derived a dataset of estimated 3' UTR sequences of all yeast genes (see Materials and methods for details). We then created an index of all sequence elements existing in these 3' UTRs, by exhaustively enumerating all k-mers. For each k-mer (where 8 ≤ k ≤ 12) the index indicates which genes contain it in their 3' UTR (see the supplementary material to this article on our website [16] for the distribution of the number of occurrences of each k-mer for different k values). Out of 4^8^+4^9^+4^10^+4^11^+4^12 ^= 22,347,776 possible k-mers, 3,833,002 (that is, 17.15%) were present in the 3' UTRs of at least one gene. In subsequent analyses we scored k-mers for their potential effects on mRNA by examination of the sets of genes containing them in their 3' UTR. k-mers were considered significant motifs if the genes assigned to them display significantly high or significantly low half-life values, or if the proteins encoded by these genes were predominantly localized in a limited set of organelles and other subcellular locations.

### A catalog of 3' UTR motifs associated with increased or decreased mRNA stability

From a genome-wide survey of mRNA half-life decay measurements, carried out in rich YPD medium [14], we collected, for each k-mer, the set of half-life values of all the genes containing it in their 3' UTR. We then scored each k-mer by computing a *p*-value (with ranksum test) on the hypothesis that the average half-life values of the genes that contain it is either significantly higher or significantly lower than the average half-life of all mRNAs in the transcriptome (the transcriptome average life time is 26.3 mins). To control for testing of multiple hypothesis we used false discovery rate (FDR) [17] with a *q*-value of 0.1 (that is, tolerating 10% false discovery). This resulted in 515 significant k-mers, of which 473 were associated with decreased half-life, and 42 with increased half-life of the corresponding mRNA. Since the FDR was set to 0.1, about 464 (0.9*515) of these motifs are expected to be true positives. In a negative control we generated 1,000 random assignments of gene sequences to half-life values and repeated the motif derivation process. In 99% of the cases none of the k-mers passed the FDR test, and in 1% of the cases only one motif passed - in sharp contrast to the 515 k-mers that passed the test in the real data.

We then checked whether the discovered k-mers probably act as single- or double-stranded motifs. While DNA motifs in promoter regions are usually expected to score as highly as their reverse complement (since binding proteins often recognize both strands), the reverse complement of RNA single-stranded motifs are not likely to be functional. Thus, unlike the common practice in promoter regulatory motifs [18], we did not unify the set of genes containing a k-mer with the genes that contain its reverse complement. Consequently, we could then test whether the high-scoring k-mers are more likely to function as single- or double-stranded motifs, that is, as motifs that function respectively at the DNA or at the RNA levels. Indeed, we found that none of the 515 significant k-mers had its reverse complement in the set of significant k-mers, suggesting that the motifs are acting at the RNA level (the motifs could not function at the protein level either, since they occur past the stop codon).

**Figure 1:**
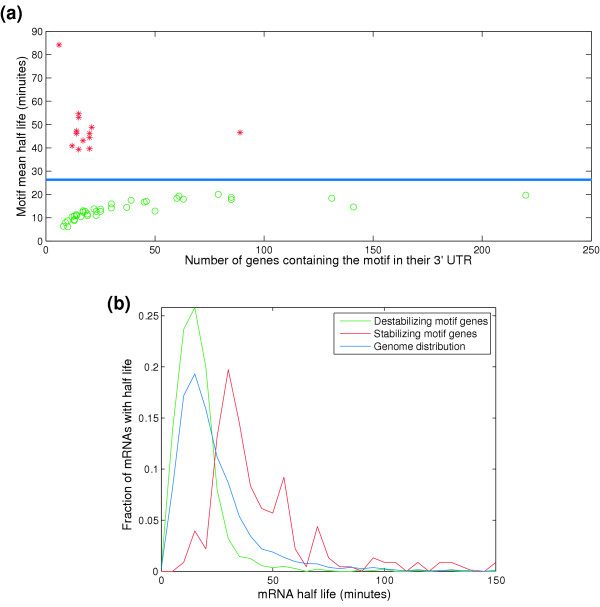
mRNA half life distributions. **(a) **The mean half-life versus gene target set size of 50 stabilization-associated 3' UTR motifs. The genome mean is indicated by a blue line at 26.3 mins. Each stabilizing motif is marked with a red asterisk, and each de-stabilizing motif is marked by a green circle. Motif M1, which mediates a mean half-life of 16 mins for a target set of 641 genes, is not displayed in the figure. **(b) **Half-life distribution of the target gene sets of all destabilizing motifs (green), of target gene sets of all stabilizing motifs (red), and of all genes (blue).

**Figure 2:**
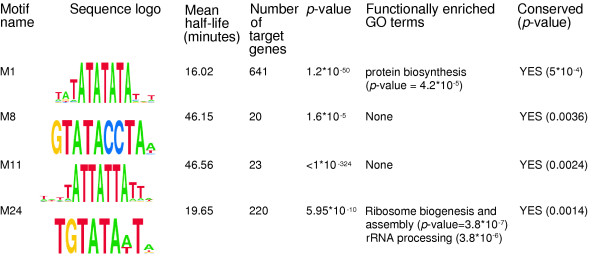
Examples of four of the 53 stability motifs discovered. M1 and M24 are destabilizing motifs, and M8 and M11 are stabilizing. Presented are mean half-life for each motif, and the *p*-value on the hypothesis that they mediate a significant increase or decrease in half-life compared with the genome, resulting from a ranksum test. Functional enrichment was tested as in Tavazoie *et al*. [5], hypergeometric *p*-values, and then applying FDR at *q*-value = 0.1. 'None' indicates that no GO term passed FDR.

**Figure 3:**
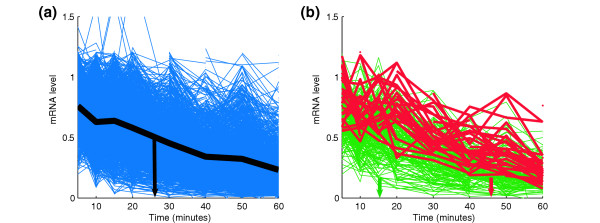
Decay profiles of the entire genome and of genes regulated by a stability and a de-stability motif. **(a) **Decay profile of the entire genome; the black curve shows the genome average profile. **(b) **Decay profiles of the target gene set of the destabilizing motif M1 (green), which has a mean half-life of 16 mins, and the stabilizing motif M11 (red), which has a half-life of 46.5 mins. The mean half-lives are marked by arrows. Expression data profiles, as well as half-lives computed using a fit to an exponential function, are from Wang *et al*. [14].

We clustered the 515 high-scoring k-mers according to sequence similarity using ClustalW [19], and merged sets of genes that are assigned to motifs that belong to the same cluster (see Materials and methods for details). With such unified gene sets we then recalculated the *p*-values on the hypotheses that they display significantly high or low half-lives, compared with the genome average. The procedure resulted in 51 clusters of motifs, each represented in the form of a position specific score matrix (PSSM). The mean half-lives of the genes associated with each motif cluster are shown in Figure 1a (see Figure 1b for distribution of half-life values for the genes containing stability-associated motifs). Several examples for such high scoring PSSMs can be seen in Figure 2; sequence logos of all PSSMs are available on our website [16]. Out of the 51 motifs, 38 were found to be associated with mRNA destabilization, and 13 are putative stabilization-related motifs, as deduced from significantly low or high average half-lives, respectively (see Figure 3 for examples). Most of the clustered motifs were found to regulate a few dozen of mRNAs (on average 32 transcripts/PSSM). A few are considerably more prevalent, the most abundant of which is motif M1 with the consensus TATATATA, which appears in 641 3' UTRs (see Figure 2). Most importantly, the functional significance of this motif was verified experimentally on the gene CYC1 [20].

In an attempt to expand the catalog further, and minimize the amount of false negatives, we then loosened the *p*-value threshold and further examined the next 500 most significant k-mers that were not included in the original set of 515 significant k-mers. In a similar fashion to [2], for each of these 500 k-mers we examined all possible degenerate forms obtainable by replacing any one or two positions in the k-mer by IUPAC symbols (see Materials and methods). Out of the 500 sets of degenerate forms of a motif, 471 had at least one degenerate k-mer with improved *p*-value relative to the original corresponding non-degenerate motif. However, a comparison of these improved k-mers with our original catalog of 51 motifs showed that all motifs (except for one which turned out to be present in retrotransposone-related genes and therefore was discarded) were found not to be sufficiently distinct (CompareACE score > 0.5) from at least one of the motifs in the original catalog, and therefore we could not consider them as new motifs.

We also utilized a complementary approach for motif discovery that is based on forming gene sets with similar half-life values, followed by a search for over-represented motifs in each gene set. For this, we used the Gibbs sampler, AlignACE [21], in a modified version that handles single-stranded sequences (see Materials and methods). We formed gene sets by grouping together genes that belong to the same percentile of the half-life values distribution. We ran the Gibbs sampler on the gene sets that constitute the top and bottom 10th, 20th and 30th percentiles of the distribution, as well as each bin of 10% separately. The search resulted in three significant motifs, one of which is almost identical to M24 (which was derived by the exhaustive k-mer enumeration procedure). M24 was found to be significantly over-represented in the 10th and 20th percentile clusters with shortest half-lives, as was also previously demonstrated by Graber *et al*. [22]. The other two motifs, marked M52 and M53, were not discovered by the k-mer indexing method.

### Using evolutionary conservation for selecting high confidence motifs

Having established a catalog of candidate motifs, we can now highlight high-confidence motifs based on evolutionary conservation information. We calculated the conservation rates of the 53 motifs in three other sequenced *sensu stricto Saccharomyces *yeast species, and also compared them with recently discovered 3' UTR motifs conserved in mammalian genomes [11]. For the conservation analysis in yeast we used data by Kellis *et al*. [23], containing the alignments of 4,919 *Saccharomyces cerevisiae *ORFs to their orthologous sequences in the three other *sensu stricto *species, along with their flanking upstream and downstream sequences, and calculated a *p*-value for the conservation rate of each of the 53 motifs (see Materials and methods). Out of 53 stability-associated motifs, 16 (30%) had a conservation *p*-value smaller than 0.05, and many more show a conservation rate that is markedly higher than the 1.85% average conservation rate of k-mers in the background 3' UTR sequence (see Figure 2 and supplementary data [16]). We note that for 10 of the 53 motifs, a large fraction (>75%) of the genes in *S. cerevisiae *do not have all three orthologs, and thus in this case conservation is not well-defined, so in fact 16 out of the 43 motifs (37%) for which conservation could be calculated are conserved.

**Figure 4:**
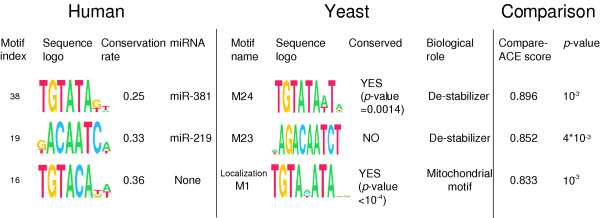
Examples of yeast 3' UTR motifs and their best mammalian counterpart 3' UTR motif. All 72 mammalian motifs were transformed into alignments and then PSSMs, and compared with all 53 yeast motifs using CompareACE [21]. The figure presents, for the mammalian motifs by Xie *et al*. [11] its motif index in the original paper, the sequence logo, conservation rate, and a corresponding miRNA which is presumed to bind the motif. For the yeast motif, the motif name, sequence logo, significance of conservation across four *sensu stricto *yeast species, and the potential biological role are shown. The CompareACE score for similarity between the mammalian and yeast motif, along with a *p*-value on it, are presented on the right-hand side of the figure.

Recently, 72 clusters of conserved 3' UTR motifs were discovered in mammalian genomes, of which nearly one half were associated with microRNAs [11]. We compared all the 53 stability-associated motifs discovered here against the 72 mammalian motifs and detected striking conservation for 10 yeast-mammal motif pairs (see Figure 4 for examples, Materials and methods and supplementary data [16] for the motif conservation information). We stress the fact that some motifs were conserved in human but not in yeast, indicating that our use of the half-life data was crucial, as conservation in yeast alone could not have detected these motifs.

Overall, 22 of the motifs in the catalog show significant conservation either within the *sensu stricto *yeast species and/or in human; these constitute 51% of the motifs for which conservation is calculable. Those highly conserved motifs thus represent our high-confidence motifs. They contain the experimentally validated M1 and M24 motifs, in addition to another motif described below. Yet, akin to the case of many verified functional motifs in yeast promoters [24], it is possible that some of the non-conserved motifs represent species-specific motifs.

### Functional analysis of the stability-associated motif catalog

We calculated a positional bias score [21], that is, a tendency of a motif to be located at a specific distance relative to the start of the 3' UTR, for all 53 motifs in the catalog. We found that 48 of the motifs have significant positional bias (with a *p*-value threshold of 0.0362 which corresponds to an FDR of 0.05). The mean preferred distance from the stop codon for these 48 motifs is around 100 nucleotides. Such positional bias is a hallmark of many promoter motifs [21] and may similarly characterize functional stability-associated motifs.

We wanted to examine next whether the relatively short motifs discovered here work in a 'context dependent manner', that is, whether their flanking sequence is constrained or not. For this, we examined windows of 20 nucleotides centered around each motif in all the genes that contain them and calculated the information content (IC) of each such position. In 14 out of the 53 motifs in the catalog we observed nucleotide positions that flank the motif whose information content value was at least as high as in the motif itself (see all 53 IC plots in our supplementary data [16]). The rest of the 44 motifs appear to operate in a context-independent manner, and a reasonable hypothesis may thus be that if inserted into a heterologous UTR they may still exert their regulatory effect. In addition, we also examined the effect of removal of less safe assignments of genes to motifs on the information content within the motif and in the flanks. For the sake of this analysis, 'less safe' assignments were defined as genes that contained in the 3' UTR an instability-associated motif, yet their half-lives were higher than the genome average, or genes assigned to a stability-associated motif whose half-life was lower than that of the genome average (we note though that it is entirely possible that these cases do in fact represent genuine assignments and the half-lives would have been even more extreme without the motifs). We filtered out these genes from each motif, and recalculated the IC profiles within the motifs and in the flanks. In several cases, we can see that the IC of positions outside the motif has increased as a result of the filtering. These positions might be functional, for example, involved in the regulatory effect of the motif, since they are more conserved in the set of genes that remained after filtration of the outliers. Another possibility is of more subtle effects by the surroundings of the motif, such as secondary structure.

**Figure 5:**
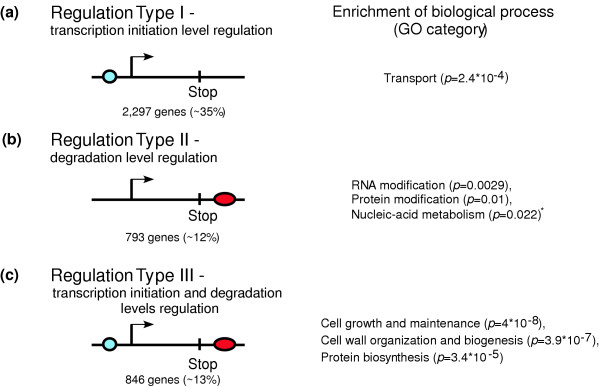
Three types of mRNA transcript regulation. **(a) **Type I: transcription initiation level regulation - genes that contain promoter regulatory motif(s) (blue circle) in their promoter according to Harbison *et al*.'s data [25], but do not contain any of the stability-associated motifs from the present analysis. **(b) **Type II: transcript degradation level regulation - genes that contain stability-associated motif(s) (red oval) from the present analysis but do not contain any of the promoter motifs from [25]. **(c) **Type III: combined transcription initiation and transcript degradation level regulation - genes that contain both promoter motif(s) and stability-associated motif(s). The figure shows the number of genes in each regulation type and the enriched biological processes that were found for them. Enrichment was calculated as a hypergeometric *p*-value using GO annotations.The enriched processes that were found significant after FDR (*q*-value = 0.1) are stated for types I and III. *In type II only borderline significance was found, (no term passed FDR) and those are reported along with their *p*-values.

We further investigated the expression of the genes that contained stability-associated motifs. We checked which of these genes contain, in addition to a putative stability-affecting motif, promoter motifs that probably exert regulation on them at the level of transcription initiation. For this purpose we used genome-wide promoter-binding data published recently by Harbison *et al*. [25], which identify yeast genes that bind to each of around 200 known transcription factors. We defined three types of genes according to different modes of their regulation: Type I: genes regulated mainly at the transcription initiation level, Type II: genes regulated primarily at mRNA stability level, and Type III: genes subject to a combined regulation at both transcription initiation and mRNA stability levels (see Figure 5). We then wanted to further functionally characterize the genes that appear to be subject to the different types of regulation. Examination of the Gene Ontology (GO) [26] biological processes that characterize genes subject to Type III regulation revealed statistically significant enrichment for several functional GO terms, including cell growth and maintenance (*p*-value = 4*10^-8^), cell wall organization and biogenesis (*p*-value = 3.9*10^-7^) and protein biosynthesis (*p*-value = 3.4*10^-5^). Genes subject to Type I regulation, which only contain a promoter motif, are enriched for transport (*p*-value = 2.4*10^-4^). *p*-values were computed using the hyper-geometric model [5], and only hypotheses that passed an FDR test with *q*-value = 0.1 are reported. On the other hand, among genes subjected to Type II regulation, which are predicted to be regulated only at the mRNA degradation level, we only found barely significant enrichments (which did not pass the FDR-requirement), for example, for 'RNA modification' (*p*-value = 0.0029), 'protein modification' (*p*-value = 0.01) and 'nucleic-acid metabolism' (*p*-value = 0.022) (see our supplementary data [16]). We note, though, that such gene classification into the three types is very preliminary since we are still far from a complete, error-free, stability motif catalog, and even the set of promoter motifs is probably incomplete.

**Figure 6:**
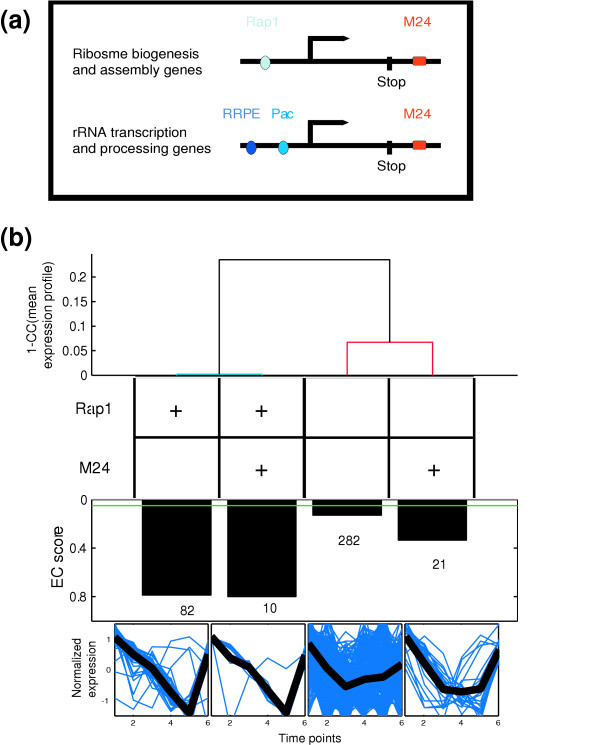
A combined regulation of protein biosynthesis genes by promoter and 3' UTR motifs. **(a) **A schematic depiction of the regulation of typical ribosomal biogenesis and assembly genes and of rRNA transcription and processing genes. While many protein biosynthesis genes (predominantly ribosomal genes) are regulated by Rap1 in their promoters, and most rRNA transcription and processing genes are regulated by the combined Pac-RRPE cassette, these two types of genes are suggested here to share a stability-associated motif in their 3' UTR, namely M24. **(b) **Combinogram analysis [18] of the protein biosynthesis genes in the condition of environmental response to peroxide stress [61]. We gathered all genes annotated with protein biosynthesis by the SGD [32] and partitioned them into four disjoint sets: genes containing only RAP1, only M24, both of them and neither of them. The motif presence is marked by a plus symbol in the second panel. The first panel presents a dendrogram built using the correlation coefficients between the mean expression profiles of each of the four sets. We also present, for each set, its EC score [18,31], in a bar diagram. All four EC scores had a *p*-value < 0.05. The number of genes in each set is also given, for which we had expression profiles in the presented condition. Finally, in the fourth panel, we show the expression profiles of the genes in each set in blue, and their mean profile in black. The genes on the far right of the fourth panel, which contain only M24 in their 3' UTRs, but not Rap1 in their promoter, exhibit a significantly more coherent behavior than the background set (genes containing neither of the two motifs) and their profiles show a sharper decrease in the beginning of the experiment.

We also tested the set of genes assigned to each of the 53 stability-associated motifs for enriched biological processes. For each of the GO biological functional terms and for each motif we calculated a *p*-value on the over-representation of the term within the set of genes with the motifs using the hyper-geometric score. Two motifs, M1 and M24, passed an FDR (*q*-value = 0.1) test for functional enrichment of specific GO-annotated biological processes (see our supplementary data [16]). Motif M1, which is hypothesized to mediate destabilization with a mean half-life of 16 mins, and which appears in the 3' UTRs of 641 genes, was found to be highly enriched for the 'protein biosynthesis' GO functional term. Motif M24, which is also predicted to mediate destabilization (mean half-life 19.4 mins, controlling 220 genes), was found to be enriched for 'ribosome biogenesis and assembly', as well as for 'rRNA processing' and 'transcription from Pol I promoter'. We note that this motif was previously discovered to be over-represented among genes with low half-lives [22], and was recently suggested as the binding site for the Puf4 protein, which is known to reduce gene expression levels by affecting mRNA stability [27]. We have previously reported [18] that ribosomal proteins and rRNA processing genes are similarly (though distinctly) expressed in most conditions, despite having disjoint promoter motifs. The observation that M24 is present in the 3' UTRs of genes belonging to both functional categories is thus intriguing since it may explain the coarse co-expression of these genes, through a potential effect on transcript stability (see Figure 6a).

Focusing on the ribosomal proteins, we found that 23 genes, belonging to the protein biosynthesis category, contain M24 in their 3' UTRs but not Rap1, a major promoter-binding regulator of these proteins [28], in their promoters. We hypothesized that the M24 motif regulates these genes in the absence of the promoter transcription factor binding sites characteristic of their functional categories. In order to check this possibility we analyzed conventional (that is, steady-state and not degradation) expression experiments in a set of 40 conditions measured across time series [29], representing a variety of natural and perturbed conditions obtained from ExpressDB [30]. In order to dissect the effect of Rap1, M24 and their combination on gene expression profiles we performed a Combinogram analysis [18], which amounts to partitioning all the genes involved in protein biosynthesis into four sets - genes that contain Rap1 in their promoter but not M24 in the 3' UTR, genes that contain M24 in the 3' UTR but not Rap1 in the promoter, genes that contain both motifs, and genes that contain none of the motifs. For each such gene set, in each expression condition, we measured the expression coherence (EC) score [18,31] (a measure of the extent of clustering of a gene set in expression space, see Materials and methods for more details), and also depicted the similarity of the expression profiles between all four sets of genes; see Figure 6b for an example with a particular growth condition (analyses of additional conditions are available [16]). We observed that in the absence of Rap1 in genes' promoters, the presence of M24 is shown to exert a significant effect on expression - mRNAs of protein biosynthetic genes that contain M24 in the 3' UTR, but not Rap1 in the promoter are significantly more coherent than the mRNAs of protein biosynthetic genes that contain none of the two motifs (of *p*-value < 10^-3^), see EC bar in the Combinogram in Figure 6b. Such effect was seen in 10 out of the 40 examined conditions (see our supplementary data [16]). Since we discovered the motif through its association with decreased stability, we propose that the significant coherence observed at steady-state mRNA level, in genes that lack Rap1, may result from concerted degradation that is mediated by the M24 motif. It is also interesting to note that protein biosynthesis genes that contain M24 but not Rap1 have an expression profile that is distinct from the typical Rap1-dictated profile of protein biosynthesis genes, yet genes that contain the two motifs behave like typical Rap1-regulated genes (see the dendrogram part of the Combinogram in Figure 6b).

### A catalog of 3' UTR motifs associated with subcellular localization

Since 3' UTRs of genes may also determine the subcellular localization of mRNAs, we next turned to identify 3' UTR motifs that are associated with particular subcellular localizations. For this, we used the k-mer enumeration method described above, but with a different scoring function: at first we used the k-mer index to find motifs significantly associated with restricted subcellular localizations, and then tried to expand the catalog by loosening the significance threshold and examining degenerate motifs, as described above. For this we used genome-wide data on subcellular localization at the protein level of yeast genes [26,32].

We introduced a measure, called subcellular clustering (SCC), which evaluates the extent to which a set of genes is expressed predominantly in one or a few subcellular locations or organelles within the cell (see Materials and methods). Altogether, 79 significant k-mers passed the FDR test (*q*-value = 0.1). Remarkably, in the subsequent clustering stage all 79 k-mers were clustered into a single motif whose consensus is TGTAHATA. The motif appears in the 3' UTRs of 610 genes, of which 260 are annotated to be localized to the mitochondria. More specifically, the motif is over-represented (*p*-value = 3.35*10^-7^) within a set of genes whose mRNAs are translated in polyribosomes that are attached to the outer side of the mitochondrial membrane [33]. Indeed the motif was identified previously in a specific search on mitochondrial genes [34] and more recently as a candidate binding site of the RNA binding protein Puf3p [27]. We also noticed that the motif has a strong positional bias (*p*-value = 1.4*10^-38^) towards the first 20-40 nucleotides of the 3' UTR. Considering that only 505 out of the 610 genes containing the motif have an annotated cellular localization, we hypothesize that some of the un-annotated genes with the motif may as well be localized to the mitochondria.

**Figure 7:**
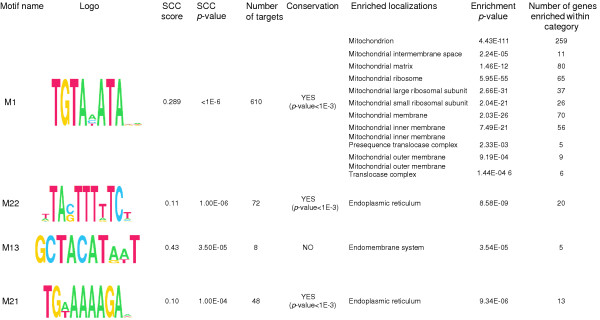
Examples of four of the 23 subcellular localization-associated motifs. Presented are motif name and logo, SCC score and *p*-value, number of target genes in whose 3' UTR the motif appears, and *p*-value for evolutionary conservation in other yeasts. Localization enrichment was computed by hypergeometric *p*-value, and only terms passing FDR at *q*-value = 0.1 are reported.

We then loosened the significance to include the next 500 most significant k-mers that were not admitted in the catalog, and examined their degenerate forms with one or two IUPAC symbols (identical to the procedure used with the stability motifs). Out of the 500 motifs, 484 had at least one degenerate k-mer with an improved *p*-value compared with the original k-mer. Interestingly, in contrast to the stability catalog where no new motif was found in this second pass, here several motifs were found to be non-similar to the above mitochondrial motif. These new degenerate k-mers gave rise to additional 22 motifs, and they were added to the catalog (see Materials and methods for more details, examples in Figure 7, and the entire catalog in the supplementary data [16]). The additional motifs display functional enrichment for various cellular localizations, such as endoplasmic reticulum (ER), endomembrane system (which is related to the secretory vesicle pathway), microtubule cytoskeleton and even the nucleus, for which a recent study indicated *in situ *translation [35]. For these motifs, we also checked the extent of positional bias and found that 13 out of the 22 have a statistically significant (*p*-value < 0.05) positional bias (see our supplementary data [16]).

When analyzing the evolutionary conservation of these 23 localization motifs in the *sensu stricto *yeasts, we found that nine are extremely significantly conserved, while one more shows a borderline significance in its conservation (see examples in Figure 7 and the full catalog [16]). More specifically, we have found the mitochondrial motif to be highly conserved in the *sensu stricto *yeasts. There are 610 *S. cerevisiae *genes that contain the motif, of which 520 were present in the dataset of orthologous yeast genes [23]. Of these, the motif is conserved in all existing orthologs in other species in 243 genes (47%; of the 243 genes, 201 genes had orthologs in all four species, and 42 genes had orthologs in three or fewer species). Such conservation has a clear functional implication: while the probability of an mRNA to localize to the vicinity of the mitochondria given that it contains the motif is 51%, this probability increases to 81% if the motif is conserved in the other yeasts (see Tables S1-S3 in our supplementary data [16]). We also note that the conservation of the sequence flanking the motif decays rapidly (see supplemental Figure S1 [16]), thus the motif is a conserved island in a region that is otherwise considerably less conserved. A comparison between this catalog and the collection of mammalian 3' UTR conserved motifs by Xie *et al*. [11] revealed that the mitochondrial motif discussed above is significantly similar to two of the mammalian motifs. The mitochondrial motif is remarkably conserved in humans - it is almost identical to both motifs #16 and #32 in the mammalian 3' UTR motif collection.

Our rediscovery of the mitochondrial motif, which has other experimental and computational evidence in the literature, is a demonstration of the validity of our method. The fact that many other motifs were found using the degeneracy method may indicate that these motifs are more variable in nature. Localization to other organelles may also be governed by secondary structure motifs, such as in the case of ASH1 [36], and can of course occur post-translationally through protein-acting motifs. In that respect the conservation of motifs at the sequence level reveals only a fraction of the actual conservation level since for some motifs only the structure may be conserved.

### Assessment of false negative rate of the method

Since we have very few known 3' UTR motifs with which we can assess the rate of false negatives of our motif discovery method, we used instead an estimation of false negative rate of rediscovery of transcription factor binding sites in gene promoters, applying the same discovery method to yeast promoter sequences (see Materials and methods for details). We found that the same methodology applied to promoter regions, using scoring functions that utilize either conventional steady-state mRNA expression profiles or GO functional annotations can rediscover up to 91% of the known transcription factor binding sites in yeast, therefore suggesting a relatively low rate of false negatives.

## Discussion

In this work, we explored functional sequence elements in the 3' UTRs in *S. cerevisiae*, and identified sequence motifs that may regulate, or at least are significantly associated with, the stability and subcellular localization of mRNA transcripts. Identification of the *cis-*acting elements that mediate stabilization or destabilization of the mRNA is crucial for understanding of mRNA degradation regulation mechanisms. In analogy to transcription initiation, where a large and probably comprehensive collection of motifs has been assembled over the years, the assembly of a parallel collection of motifs that control mRNA degradation is thus clearly of great interest.

The motifs in the present catalog were found to be correlated with significantly high or low half-life values. In addition, evolutionary conservation of a large proportion of them probably indicates that many of these motifs are indeed biologically functional. Based on conservation analysis of the motifs, and taking into consideration that some motifs may be species-specific [24], we estimate that the false-positive rate of the method is below 50%, and the prioritized set of conserved motifs probably has the least fraction of false positives. Nonetheless, at this stage many of the motif-to-gene assignments proposed here represent correlations that need further experimental corroboration, just as it is with most promoter motifs that are still mainly discovered computationally. We thus anticipate that this preliminary catalog of motifs will be followed by other computational and experimental works, which will in the future assemble a comprehensive catalog, akin to the one published recently for promoter motifs [25]. In this respect, we note that it is most likely that our approach did not discover the full set of functional stability-affecting and localization motifs in the genome. The very limited prior knowledge about stability and localization motifs in yeast precludes comprehensive assessment of the false negatives rate, although most of the few known motifs were rediscovered here, including members of the Puf family: Puf3p, Puf4p and Puf5p [27]. Puf3 is in fact the present mitochondrial motif, and Puf4 is the de-stabilizing motif M24. Puf5p was proved experimentally to bind to the TTGT sequence [37], present in several of our motifs, and was recently suggested as an expanded sequence by Gerber *et al*. [27] and is most similar to the present M15. In addition, the functional significance of M1 was validated in the 3' UTR of the CYC1 gene by Russo *et al*. [20]. On the other hand, the localization motif on ASH1 [36], which was shown to be a secondary structure motif, was not discovered by our study, as it focuses on sequence motifs. As a complementary means of assessment of the rate of false negatives we checked our ability to rediscover promoter motifs from a well-established set [25] using the same k-mer indexing method, with a scoring function that assesses the effect of promoter motifs on steady-state mRNA expression profiles of downstream genes (the expression coherence and its *p*-value [18,31] and the functional coherence score and *p*-values, see Materials and methods). Using the EC score we found that up to 91% of the known transcription factor binding motifs are blindly rediscovered by the indexing method, suggesting a good coverage, or low false-negative rate of the procedure (see Materials and methods for details). We note, however, that steady-state mRNA expression data are available, and were used for this coverage assessment, in several natural and stressful growth conditions, while decay profiles are currently available only in rich medium. We thus estimate that the full potential of the method to discover functional 3' UTR motifs will be fulfilled when mRNA decay profiles become available in additional growth conditions. With GO annotations, a smaller proportion, 44% of the known motifs, are rediscovered. Yet this result is by itself encouraging, as it suggests that there is sufficient information in functional annotations to rediscover almost a half of the motifs gathered so far in this heavily studied organism, indicating that our GO-based 3' UTR motif discovery, applied here for the subcellular localization motifs, may also cover a significant proportion of the existing functional motifs in these regions.

Evolutionary conservation information was utilized in this motif discovery process *a posteriori*, that is, candidate motifs were identified based on expression/subcellular location information and then their conservation was evaluated later as a means of prioritization. We thus primarily stress the functionality of the motif, allowing in principle the discovery of species-specific motifs. As an alternative, conservation information could be used as an *a priori *stage, that is, conserved 3' UTR elements could be identified and a search could then be carried out, for example, in the form of the present ranksum-based test, which assess the functionality of the motifs. In this alternative direction the emphasis is on high conservation and future work will be needed in order to compare the two approaches.

The scope of the current work was intentionally restricted to 3' UTRs since these regions have been implicated before in message stability and localization [38-43]. Yet it is still entirely possible that other regions, such as the 5' UTRs and the coding regions, may contain motifs that control stability and localization. However, the analysis of these regions is much more complex, since regulatory motifs may be intricately intertwined with protein motifs, and may be affected by amino acid or codon biases in the case of coding regions, and with promoter motifs in the case of the 5' UTRs. Indeed, most studies that looked for promoter motifs have consciously included the 5' UTRs and many transcription motifs are found in proximity to the ATG, that is, most probably within the 5' UTRs. Future analysis of those regions will have to account for all the above in order to disentangle stability and localization affecting motifs from other sequence signals.

At the first stage of our motif discovery process we employed two alternative types of algorithms in parallel: exhaustive k-mer indexing and discovery of over-represented PSSMs in gene sets clustered by half-life values. While the latter approach is more prevalent in promoter-motif finding [5,44-46], several works used the k-mer-based approach, see, for example [2,47]. Recently, a comparison of prevailing motif finding algorithms concluded that a k-mer based method [48] outperformed the others [3]. In our case, the k-mer-based approach allowed us to examine the entire space of fixed k-mers and to sample degenerate k-mers, and it indeed resulted in many more significant motifs. Many of the motifs discovered in the k-mer approach only are clearly not over-represented in particular bins of half-life values. Moreover, many of the motifs in the catalog are present in the 3' UTRs of a relatively small number of genes, whose half-life values may be similar to those of other genes that lack the motifs, a situation that precludes the possibility of their discovery through a contemporary over-representation-based algorithm. For example, M32 is located in the 3' UTR of only six genes, but is associated with an extremely high mean half-life of 84 mins, and thus was found to be highly significant. This motif is not over-represented in any cluster of genes and thus could not and was not discovered by algorithms such as AlignACE.

The 3' UTR-mediated effects on mRNA stability on one hand, and on subcellular localization on the other hand, appear to be at least partially overlapping with two respective well-known mechanisms. mRNA steady-state levels are also largely affected by transcription initiation rates, and subcellular localizations are often determined through protein targeting signals. We note that among the 652 annotated mitochondrial genes, 260 have the present RNA motif and 362 have the protein mitochondrial targeting signal (MTS) [49], where 160 genes have both signals and 190 have none. What appears to complement protein-level trafficking to the mitochondria is a mechanism in which mitochondrial proteins are translated *in situ*, that is, in the vicinity of the organelle [50-52] in a process of co-translation import [53-55]. The mitochondrial motif that we identified, which was suggested to be the consensus binding site of Puf3 [27], could facilitate the above co-translational import mechanism by directing mRNAs to the polyribosomes. However, we note that Huh *et al*. [56] have reported the localization of a large fraction of the nuclear-encoded mitochondrial proteins to the mitochondria, even without their 3' UTRs. This might suggest that the mitochondrial mRNA motif presented here is redundant to protein localization signals that may enhance the efficiency of the localization process. In general, there may be subtle roles of mRNA localization, which seem to be partially redundant to protein localization mechanisms that are not yet completely understood. The identification and characterization of the motifs that are associated with mRNA localization may help to further understand such roles.

The k-mer indexing method and the scoring method introduced here can be generalized. For instance, we have previously shown that a similar scoring function can be used to dissect promoter motifs and the effects of substitutions within them on gene expression [31]. In the future, catalogs of sequence motifs that regulate other processes and properties, such as translational efficiency and protein abundance levels, may be discovered using a similar methodology provided that appropriate data are available.

The control of mRNA steady-state level is obtained through promoter regulatory motifs that affect transcription initiation. Why is there a need to influence mRNA steady-state level at the degradation level too? The most likely effect of controlled degradation is a sharper decrease in the message steady-state level when needed, compared with the delay that would occur if such a decrease were to be obtained merely by halting transcription initiation (Shalgi *et al*., work in progress). It was noted however, that protein degradation-based regulation has the metabolic cost of 'futile production' [57]. In that respect our discovery of the potential ribosomal mRNA degradation motifs (M1 and M24) is particularly interesting. On one hand these gene products are under very tight regulation and the mechanisms to shut them down have to be very fast. A parallel degradation of these products at the mRNA level may have the advantage of reduced futile production cost, in comparison with protein-level degradation, especially in view of the huge amount of translated ribosomal proteins in cells. In addition, it was recently suggested [58] that due to the accuracy of promoter-based regulation, microRNAs may function as 'micromanagers' that can tighten mRNA level regulation. But whether yeast species utilize this particular mechanism, or alternative ones to achieve this regulatory task, remains to be explored.

## Conclusion

We present here two novel catalogs of functional motifs in 3' UTRs: a catalog of 53 stability-associated motifs and a catalog of 23 subcellular localization motifs. Although in the derivation of the motifs only half-life and localization data were used, many of the motifs showed, *a posteriori*, three important properties: high evolutionary conservation, high positional bias, and, in the case of the stability motifs, their presence or absence in 3' UTRs was correlated with different steady-state levels of mRNAs. The discovered motifs should thus be instrumental in complementing promoter motifs in modeling of the transcriptome.

## Materials and methods

### The set of yeast 3' UTRs

Since the 3' UTRs of *S. cerevisiae *are not well annotated, the set of 3' UTRs was built based on the *S. cerevisiae *mRNA length data of Hurowitz and Brown [15]. This work determined the total length of mRNAs for most *S. cerevisiae *genes, and by subtraction of the ORF length from the mRNA length, we calculated the total length of the UTRs, 5' UTR plus 3' UTR. The total length was used for the 3' UTR sequence extraction. For the 1,823 genes not covered by Hurowitz and Brown, we used the average of the UTR length (300 bp). Thus our set of putative 3' UTR is maximalistic in that sense, as we preferred to err on the side of having more, rather than fewer, of actual 3' UTRs. In reality, we found that most significant motifs occur on the first 100 bases downstream of the stop codon (see Results). 3' UTR boundaries derived from EST libraries may serve as an alternative, yet they are available for only about 900 genes in the genome [59].

### Half-life dataset

mRNA half-life data were taken from Wang *et al*. [14]. We used the data version generated with random priming, termed 'overall decay' in [14], and employed the 'external control' method that utilizes *in vitro *synthesized RNAs for control. The microarray measurements in the experiments were done in triplicate to assure reproducibility. Due to late time points being given equal weight in the half-life calculation procedure, some half-life values of short-lived mRNAs may have been originally under-estimated. The effect of such potential error on the present motif finding could be assessed in the future with improved stability measurements.

### Clustering of significant k-mers to motifs

All significant k-mers were clustered by their sequence similarity using ClustalW [19]. The ClustalW step resulted in an alignment of each cluster. After that, for each k-mer within a cluster, we went back to the 3' UTR sequences of the k-mers genes, and filled the gaps in the alignment with the original sequence, so that finally we had, for each cluster, a file containing all the sequences of all the occurrences of the k-mers within it, and these files (which are available in our supplementary data [16]) were used to generate the PSSM and the sequence logo. This way, each nucleotide in each position was weighted in a way that is proportional to the actual number of its occurrences in the entire set of 3' UTRs.

After clustering, each cluster constitutes a motif, and is assigned with the set of genes that originally belong to the k-mers constituting it. We calculate for each motif the mean half-life and stability ranksum *p*-value. All motifs whose *p*-values were above the maximum *p*-value of the original collection of significant k-mer (4.8*10^-5^) were refined as follows: we removed from the set of genes assigned to a instability-associated motif all genes whose half-life values exceeded that of the genome average, and from the set of genes assigned to a stability-associated motif we removed all genes with half-life value smaller than that of the genome average.

### Clustering half-life data and finding over-represented motifs

We binned the genes according to ranked half-life values in three different ways: ten equally-populated quantiles; the highest and lowest 20th percentiles; and the highest and lowest 30th percentiles. We then ran the Gibbs-sampling based motif finder, AlignACE [30], on the 3' UTRs of genes in each bin in order to detect over-represented motifs. Since AlignACE was originally designed for promoter motifs that probably bind double-stranded DNA, we modified the algorithm to look for single-stranded motifs only, as these were supposedly RNA motifs. We retained motifs with MAP score higher than or equal to 10 [30], and group specificity *p-*value smaller than 10^-4 ^(a threshold looser than that used in [30] was chosen here in order to have sensitivity towards even very weak motifs). Furthermore, we filtered out, by manual inspection, motifs that resulted from a family of paralogs present in the first cluster of 10% lowest half-life genes, with identical UTRs.

### Expanding the catalog with degenerate motifs

A potential source of false negatives of the k-mer enumeration method is that some functional motifs may have degenerate positions such that each of their exact k-mer alternatives that correspond to their degeneracy is by itself not statistically significant. This may require enumeration and scanning with degenerate motifs. Yet since the alphabet of all IUPAC symbols consists of 15 symbols it is computationally unfeasible to exhaustively scan all possible degenerate k-mers for k values in the present range. We thus adopted an alternative procedure that would allow limited examination of promising degenerate k-mers. In each of the two catalogs, using the list of all possible k-mers that are sorted by the respective *p*-values, the procedure began with the 500 k-mers that immediately follow the most significant motifs (that is, those that passed the FDR test). Then, from each of these 500 barely significant k-mers, we generated a set of degenerate k-mers each with up to two IUPAC symbols, which may appear anywhere in the motif. The number (N) of degenerate k-mers generated from each of the 500 barely significant motifs is calculated (11 is the size of the IUPAC alphabet without the four nucleotides):



Typically, N ranges from approximately 3,000 degenerate forms per one exact 8-mer, to approximately 8,000 degenerate forms per one exact 12-mer. In total we examined around 3 million degenerate motifs in each catalog. Each degenerate motif was scored with the same methods, namely, SCC *p*-value in the case of the subcellular localization, and stability ranksum *p*-value in the case of the stability catalog. Then, for each of the 500 k-mers we chose its degenerate form with the best *p*-value out of all N degenerate motifs, and examined whether this best motif has a *p*-value better than the *p*-value of the original, non-degenerate k-mer. Only k-mers that were improved by their degenerate form were picked for subsequent analysis.

Then these motifs were compared with the original PSSMs in the catalog using CompareACE (similarity distributions can be viewed in our supplementary data [16], see Figure S2). Only motifs which had a maximal CompareACE similarity score smaller than 0.5 were considered new motifs and were included in the catalog. A CompareACE cutoff of 0.5 was taken to ensure that we added only novel motifs to the catalog, and not variants on motifs which were already discovered in the first step of the exact k-mer method.

### Adding degenerate motifs to the subcellular localization catalog

Out of the improved motifs which resulted from degenerate motif examination, only motifs found to be non-similar to the mitochondrial motif, with a CompareACE score smaller than 0.5 (see supplementary data, Figure S2b [16]) were subjected to further analysis. We further checked those motifs for GO functional enrichment in the cellular component, and filtered out motifs in one of three cases: if they were not associated with any specific organelle; if they had no enrichment at all; or if they were found exclusively in a family of paralogs. In addition, the remaining 27 degenerate motifs were clustered among themselves according to sequence similarity (as measured by CompareACE) using hierarchical clustering, and resulted in additional novel set of 22 motifs.

### Evolutionary conservation within *sensu stricto *yeasts - rates and *p*-values

The conservation rate of a motif was defined as the fraction of its occurrences in *S. cerevisiae *which was perfectly conserved within the multiple alignment of the 3' UTRs of the orthologous genes in the rest of the *sensu stricto *yeasts. We calculated three rates for each motif: conservation in all four species, in three or more, and in two or more. In order to assess statistical significance of rates of conservation we used 1,000 randomly picked distinct 8-mers from the *S. cerevisiae *set of 3' UTRs as a negative control, and checked their conservation using the same criteria. As an empirical *p*-value on the hypothesis that a motif shows a level of conservation expected for a random 3' UTR sequence, we calculated the fraction of the random 8-mers that are more conserved than the examined motif. In this negative control the mean observed conservation rate for four species was 1.85%, for three or more species 7.53% and for two or more species 23.76%. We report all motifs that received a *p*-value less than 0.05 in either of the three options.

We note that for those stability motifs, of which a large fraction (>75%) of their target genes in *S. cerevisiae *do not have all three orthologs, we verified that their genes are not in the list of spurious ORFs in [23].

### Comparison between the sets of yeast and human 3' UTR motifs

The set of 3' UTR motifs in humans was taken from the recent publication of Xie *et al*. [11], in the form of clusters of 8-mers. The 8-mers in each cluster were aligned using ClustalW [19], and the gaps were filled with nucleotides with equal frequencies to create PSSMs. Then, a similarity score for each human and yeast PSSM was obtained using CompareACE [21]. For each human 3' UTR motif, a best match from the yeast set was selected. A *p*-value on the significance of the best match was derived from a distribution of best match scores created through comparison of the human set with 1,000 reshuffled yeast motif sets.

### Expression coherence score

The expression coherence (EC) [18,31] score is a measure of the extent of co-expression of a set of genes. Given the expression pattern of each gene in the set, the EC score is the fraction of gene pairs whose expression patterns are significantly correlated with each other, out of all possible gene pairs in the set. As a control, random sets of genes of the same size as the original set are sampled from the genome, and their EC score is calculated. A corresponding *p*-value for the original set is the fraction of random sets with a higher EC score.

### The SCC score and *p*-value

Subcellular clustering (SCC) is a term used to describe the extent to which a set of genes is similarly localized in the cell. The data on subcellular localization were derived from the GO database [26], which defines the hierarchy of functional annotations, and the gene annotation itself was taken from the *Saccharomyces* Genome Database [32]. Similarity measured between functional annotations in GO was taken to be the 'semantic similarity', defined by Lord *et al*. [60], and given the semantic similarity scores between each pair of GO annotation terms, the similarity score between a pair of genes was defined as follows:



The SCC score of a set of genes is defined as the fraction of all 'significantly similar' pairs of genes out of all pairs of annotated genes in the set, where significantly similar is a score above a threshold *θ*:



The threshold *θ *was calculated to be the 95th or 90th percentile scores of the distribution of all the pairwise similarity scores of the yeast genome. Genes without annotations, or annotated as 'cellular component unknown' were excluded from the analysis.

The SCC *p*-value was calculated using random sampling. For each set size ranging from 3 to 100, a million sets of genes were randomly picked and a distribution of SCC scores for the specific set size was derived. The SCC *p*-value of a given gene set is the number of random sets with an equal or higher SCC score. For set sizes larger than 100, an upper bound estimate was given as a *p*-value, using the distribution of scores of random sets of size 100.

### Assessment of false-negative rate using known promoter motifs

Since very few 3' UTR motifs are known we could not directly assess the false negative, or coverage rate of our method using such known motifs. Thus, in order to get an estimate for the potential coverage of the current k-mer enumeration method we instead estimated our ability to rediscover known promoter motifs from a comprehensive dataset of 102 transcription factor binding sites (TFBSs) [25]. We used the same k-mer enumeration method, but applied to all yeast promoters (promoter regions were defined as in [18]). Unlike the 3' UTR motifs that are expected to work at the RNA level and hence were defined as single-stranded motifs, the promoter motifs function at the DNA level, that is, as double-stranded motifs. Hence, the list of promoters containing each k-mer was unified with the list of promoters containing its reverse complement. This resulted in a set of genes assigned to each k-mer. We used two alternative scoring functions in parallel, the first based on expression data and the second on GO annotations. The effect of a k-mer on the expression profiles of these genes was assessed using the previously defined [18,31] EC score of the gene set under a variety of natural and perturbed conditions (Lapidot *et al*., manuscript in preparation. See our website [16] for more details). A k-mer was considered significant if the *p*-value on its EC score passed an FDR threshold of 0.1, as in the 3' UTR motifs. Altogether, 8,610 k-mers passed this FDR threshold. To estimate the coverage of the Harbison motif set, we scanned each of the 8,610 k-mers against the 102 Harbison PSSMs, applying a scoring method that assesses how likely a given k-mer is to be generated by a given PSSM. For each pair of known PSSM and k-mers we summed up the frequencies corresponding to the nucleotides observed in the k-mer, over all PSSM relevant positions. This score was then scaled to the range [0-100] by subtracting the minimal possible score that may be obtained from the PSSM (that is, the score obtained for a k-mer that corresponds to the least frequent position in each column of the PSSM) and dividing by the range of possible scores (obtained after additionally identifying the maximal possible scoring k-mer from that PSSM). Depending on the cutoff used, between approximately 16% to 50% of the 8,610 significant k-mers participate in covering between 91% to 99% of the Harbison PSSMs, respectively. We then clustered all the significant k-mers that were assigned to each TFBS and generated PSSMs from them. We then compared these PSSMs with the Harbison motif set, this time using CompareACE. At a CompareACE similarity cutoff of 0.7, we report that 80 Harbison PSSMs are rediscovered. A supplementary table on our website [16] displays all of the Harbison motifs with the corresponding motifs rediscovered here. Three points are worth mentioning: our method has a low false-negative rate as it rediscovered most known motifs; two of Harbison's motifs (GAL80 and PUT3) are represented by long (17 and 18 positions, respectively) and gapped PSSMs, and we had no match for these motifs, because we only scanned ungapped sequences of length 7-11; and most significantly, the motifs we discovered were often longer than the Harbison motifs and they provide potential extensions to the known motifs with information-rich positions.

In parallel to expression-based TFBS motif discovery, we also used GO annotations to examine our ability to rediscover known TFBSs. This was done by a scoring function that is identical to the SCC function above, yet using the 'biological process' GO annotations instead of the 'cellular component' terms. We thus term the scoring function 'Functional Coherence' (FC) due to its resemblance to the EC score. The FC analysis applied to biological process annotations for TFBS discovery is based on the widespread assumption that genes belonging to similar biological processes are co-regulated at the transcription level. Using this methodology of k-mer scoring, with the same *p*-value estimation method and FDR threshold of 0.1, we discovered 350 significant k-mers, which were clustered into 72 PSSMs. Using CompareACE with the same cutoff to compare these motifs with the Harbison set of PSSMs, we rediscovered 45 of the 102 known motifs (see [16] for complete details).
